# Medicine

**Published:** 1907-09

**Authors:** I. Walker Hall


### Progress of the Medical Sciences.

### MEDICINE.

For this summary of **the pathogenic streptococci** we are indebted to Dr. I. Walker Hall, Professor of Pathology, University College, Bristol, and Pathologist to the Bristol Royal Infirmary.

It has always been difficult to satisfactorily classify pathogenic streptococci. Morphological characters, such as the size of the cocci and the length of their chains, are insufficient. The several staining reactions are not precise enough for accurate differential diagnosis. Apart from broth, the ordinary culture media do not yield information which permits useful deductions. Even the pathogenicity of the organisms may render but little help, since their virulence is easily lost during cultural or other methods, or altered, or raised, by passage through susceptible, or non-susceptible, animals. Serum reactions have also failed to distinguish distinctly between the various types, a fact which will be well appreciated by those who have used anti-streptococcus serum.

An investigation made, however, by Mervyn Gordon[1] upon the chemical powers, or metabolic reactions, of streptococci, has opened up a new field of much promise. As a result of a number of tests of the action of streptococci on various substances, he has found that the following may be employed for diagnostic purposes :—

Two dissaccharides : saccharose and lactose.
One trisaccharide : raffinose.
One polysaccharide : inulin.
Two glucosides : salicin and coniferin.
One alcohol : mannite.

These, together with the clotting of milk, and the reduction of neutral red under anærobic conditions, constitute a series of tests which point to the type of organism involved. They are not infallible, and the individual organisms exhibit considerable variations, but they permit conclusions within certain limits.

Staphylococci also may be differentiated by other tests of a similar nature, but the same precautions must be observed in making the deductions. No hard and fast line is permissible.

From a correlation of all the available records (about 1,200 strains) Andrewes and Horder[2] propose the following classification. Each type may be divided into a number of sub-types or variants :—

[1] Gordon, "Report to Local Government Board," 1906.

[2] Andrewes and Horder, *Lancet*, 1907, i. 167.

1. **Streptococcus equinus,** derived chiefly from air, dust, horse-dung, &c. Generally non-pathogenic.
2. **Streptococcus mitis.** Found in human saliva and fæces. Occasionally pathogenic.
3. **Streptococcus pyogenes.** Pathogenic.
4. **Streptococcus salivarius.** Occurs in saliva and intestine. Pathogenic.
5. **Streptococcus anginosus.** Isolated from " sore throats." Pathogenic.
6. **Streptococcus fæcalis.** Occasionally pathogenic.
7. **Pneumococci.** Pathogenic.

The reactions of these organisms are stated in the following table (Andrewes and Horder, ix.) :—

| Types. | Milk clot. | Neutral red. | Saccharose. | Lactose. | Raffinose. | Inulin. | Salicin. | Coniferin. | Mannite. | Growth at 20° C. | Morphology. |
|---|---|---|---|---|---|---|---|---|---|---|---|
| Streptococcus pyogenes | . | . | + | + | ... | ... | ± | ... | ... | + | Longus. |
| ,, salivarius | + | ± | + | + | ± | ... | ... | ... | ... | ± | Brevis. |
| ,, anginosus | + | ± | + | + | ... | . | ... | ... | ... | ± | Longus. |
| ,, fæcalis | + | + | + | + | ... | ... | + | + | + | + | Brevis. |
| Pneumococcus .. .. | ± | ... | + | + | + | ± | ... | .. | ... | ... | Brevis. |

It is of interest to consider the relation of diseased processes to the particular type of streptococcus. The following figures are taken from the papers available :—

1. **Suppuration.**

Streptococcus pyogenes .. .. .. .. 30 cases.
,, salivarius .. .. .. .. 5 ,,
,, anginosus .. .. .. .. 8 ,,
,, fæcalis .. .. .. .. 2 ,,
Pneumococcus .. .. .. .. .. .. .. 19 ,,

2. **Cystitis.**

Streptococcus fæcalis .. .. .. .. 2 cases.
,, ,, (variants) .. .. 2 ,,
,, salivarius .. .. .. .. 2 ,,

Sometimes the streptococci were present alone, sometimes they were associated with B. Coli. It is quite evident that the organisms were intestinal in origin. The streptococcus pyogenes has not been met with in this condition.

3. **Erysipelas and Cellulitis.**
Streptococcus pyogenes alone. No other forms.

4. **Serous Effusions.**

| | | |
|---|---|---|
| Streptococcus | pyogenes .. .. .. .. | 3 cases. |
| ,, | anginosus .. .. .. .. | 1 case. |

5. **Non-suppurative peritonitis,**

| | | |
|---|---|---|
| Streptococcus | pyogenes (variants) .. .. | 4 cases. |
| ,, | salivarius .. .. .. .. | 3 ,, |

6. **Septicæmia.**
(*a*) *Puerperal.*

| | | |
|---|---|---|
| Streptococcus | pyogenes .. .. .. | always. |

(*b*) *Following primary streptococcal infection.*

| | | |
|---|---|---|
| Streptococcus | pyogenes and variants | 13 cases. |
| ,, | salivarius .. .. .. | 1 case. |
| ,, | anginosus .. .. .. | 1 ,, |
| ,, | fæcalis .. .. .. | 2 cases. |
| Pneumococcus | .. .. .. .. .. | 7 ,, |

(*c*) *Following infections not primarily streptococcal.*

| | | |
|---|---|---|
| Streptococcus | pyogenes .. .. .. | 7 cases. |
| ,, | salivarius .. .. .. | 5 ,, |
| Pneumococcus | .. .. .. .. .. | 2 ,, |

7. **Malignant Endocarditis.**

| | | |
|---|---|---|
| Streptococcus | pyogenes .. .. .. .. | 2 cases. |
| ,, | salivarius .. .. .. .. | 11 ,, |
| ,, | anginosus .. .. .. .. | 6 ,, |
| ,, | fæcalis .. .. .. .. | 4 ,, |
| Pneumococcus | .. .. .. .. .. .. | 1 case. |

The preponderance of intestinal and fæcal organisms in this condition is very striking, and suggests that in these patients the low virulence of the organisms may account for the defective "resistance."

In **scarlet fever** the results so far are not sufficiently decisive.

In **acute rheumatism** the organisms isolated appear to give the same reactions as the streptococcus fæcalis. Of course this, or another organism, may be etiologically connected with the disease, but it first must be proved that the organisms already isolated have not been those of "terminal" rather than "causative" infections.

The importance of these results will be appreciated in connection with treatment. It is obvious that the organism must not only be isolated from the lesion, but must be run to earth. Its precise identification is most valuable for prognosis and treatment.

If the indications are for serum treatment, then it is evident that in a serum prepared from organisms similar in type to those isolated from the lesion lies the hope for success.

Should it be necessary to prepare a vaccine from the isolated organism, then the dose will be regulated by a knowledge of the average virulence of the particular type it belongs to.

Investigations which are now in progress point to similar developments with regard to the several types of staphylococci.

**Pernicious Anæmia.**[1]—The chief points of interest brought out in the discussion on pernicious anæmia are the need for a more systematic classification of the disease, and the necessity for reviewing the condition from the standpoint of the total volume of the blood and its total quantity of hæmoglobin. It has been for some time evident that an extended knowledge of the ætiological factors concerned is absolutely necessary. The attention has been focussed on blood films and blood changes quite long enough; abnormal intestinal processes have been boomed perseveringly without being sufficiently identified; the experimental pathologist must now take up the work. The discussion was opened by Hunter, who at once emphasised the differences between Addison's idiopathic anæmia and Biermer's progressive pernicious anæmia. The latter condition includes a number of different forms of anæmia, while the former is an infective hæmolytic anæmia which is precise in type and distinct in its symptoms. The blood changes in Addison's anæmia are those resulting from hæmolysis and the subsequent regeneration of the broken-down elements. Hence we hear of normoblasts and megaloblasts, as well as of microcytes and macrocytes, far too much being made of the megaloblastic appearances. In addition, the leucocytes are increased, this feature being most marked in the bone marrow, where coarsely granular myelocytes are very abundant. Both the blood and the bone marrow changes, however, vary in extent and in their relations to each other. So far as we at present know, it is impossible to place any definite reliance upon these factors. Among the infective lesions, attention is directed to an exudative, or proliferative, glossitis. To the naked eye the tongue is dry, and generally presents one or more darkly-tinged areas. Certain gastric and intestinal lesions also occur, and *post-mortem* exhibit the appearances of localised degenerative inflammation of the mucosa, and proliferative inflammation of the submucosa.

Discussing the causation of pernicious anæmia, Hunter observed that the ordinary causes of anæmia were insufficient to produce the whole features of the disease. Infection and injection with specific hæmolytic agents alone explained the usual symptoms; the importance of this conception was realised when antiseptic treatment was adopted as a routine measure.

[1] Impressions of a discussion at the Annual Meeting of the British Medical Association, 1907.

In some cases Hunter demonstrated the presence of streptococci in the tissues, and this indication for serum treatment was followed with success; but whether he was dealing with a primary coccal infection, or a secondary invasion of the organism in debilitated tissues, was not quite clear.

Gulland pointed out that prolonged blood destruction does not give rise to pernicious anæmia, for the condition precedes the active hæmolytic stage, and thus the toxin may interfere with the formation of the blood elements rather than promote their destruction. There may even be a "predisposition" on the part of some individuals to this action on the formation processes. Leucopenia is often a distinct feature, while the increased number of myelocytes in the bone marrow is a well-known fact. He then compared the appearance of megaloblasts in pernicious anæmia with the constant presence of megaloblasts in the early embryonic life of mammals, and suggested that the increase in the red marrow in pernicious anæmia may arise from a slow formation of megaloblastic tissue, or that, when formed, the life of the tissue may be a short one only.

Lorrain Smith suggested that more attention should be paid to the changes in the spinal cord. Those at present known consist chiefly of advanced sclerosis of various tracts (the segments corresponding to the abdominal area). Much might be learned from an examination of an early case. He also drew attention to the "simulating" forms of anæmia. Malaria is sometimes accompanied by an anæmia of a "pernicious anæmia" type, as well as a secondary anæmia. An ulcerating fibroma of the intestine was accompanied by the signs of pernicious anæmia, and malignant growths are frequently associated with a similar condition.

A most valuable means of determining the extent of the hæmolysis is afforded by the estimation of the total volume of the blood, and its total hæmoglobin contents, by the Haldane-Lorrain Smith method. In fact, it is a question whether the ordinary methods of hæmoglobin estimation throw any real light on the situation. Lorrain Smith examined seven cases of pernicious anæmia, and found that the total amount of hæmoglobin was only 50 per cent. of the normal (in chlorosis it reaches 75 per cent. of the normal, and after the hemorrhage of gastric ulcer there is only 15 per cent. loss from the normal amount). The amount of blood plasma is also an important factor, for œdema of the tissues is a constant feature in pernicious anæmia. The volume of the blood is often 60 per cent. above the normal; a reduction to the normal is a favourable symptom. The fall in the volume is generally accompanied by an increase in the percentage increase of hæmoglobin. This alteration in the volume may be regarded as a distinct effect of the toxin.

Muir observed that the toxin evidently possessed independent characteristics, for although *bothriocephalus latus* and *saponin* acted on the tissues so as to induce megaloblastic changes, yet they did not entirely reproduce the appearances of pernicious anæmia. The bone-marrow changes should, however, be regarded as the expression of an injury rather than of a reaction, and of an injury which might be recovered from.

Hutchison recorded the fact that he had never met with a case of pernicious anæmia in children. This is of importance in connection with the ætiology of the disease. The marrow and blood-forming organs of the child are generally working at high pressure in order to meet the needs of the growing tissues, and it is easy to suppose that they might exhibit some tendencies to pernicious anæmia. But this is not the case. The only conditions in the child at all comparable to pernicious anæmia are perhaps those of the acute purpuric conditions of childhood. How can this difference between youthful and adult tissues be explained ?

Melland described several cases which presented the characteristic microscopic features of pernicious anæmia without any glossitic, gastric or intestinal symptoms.

Walker Hall stated that the general nutrition in pernicious anæmia shows wide variations. The extent of the variations exceeded the individual factor, and suggested that in the " symptom complex," termed pernicious anæmia, we are dealing with the actions of different types of toxins. He emphasised the need for systematic classification. In detailing the abnormal changes of metabolism, he pointed out that protein retention was a common feature, and that the manner of the protein decomposition suggested renal changes, which deserved recognition during treatment. The cells became exhausted and fatty because of the stress of work in obtaining the necessary amount of oxygen from the diluted plasma. The output of amino acids and of lactic acid required investigation by the newer methods.

The secretion of hydrochloric acid is affected. Subacidity, or absence of hydrochloric, frequently occurs. The gastric secretion exhibits close relationships with the anæmia, being increased or diminished as the anæmia improves or changes for the worse.

The distribution of iron in pernicious anæmia varies considerably. Sometimes met with in the liver, at other times it may occur in both liver and kidney, or in the kidney alone. These peculiarities are difficult to explain.

A few cases which presented all the clinical appearances of pernicious anæmia revealed at the autopsies a number of isolated tubercles in the mesentery, or in the mesentery and liver.

Boycott dealt with the anæmias associated with animal parasites. He pointed out that the two distinct species of ankylostoma duodenale produced identical symptoms of disease, although their geographical distribution was so different. The fact that the subjects of "parasitic" anæmia suffer more from the anæmia itself than from the position of the parasite in the tissues, suggests that similar conditions might obtain in the other anæmias. It is remarkable that patients with ankylostomatous anæmia possess much more physical energy than those with pernicious anæmia when the hæmoglobin percentages are about the same; in other words, the loss of hæmoglobin is felt less by the ankylostomatous, than by the pernicious, anæmia patient. Ferguson made a similar statement with regard to the Egyptian fellah, who will do a hard day's work when the hæmoglobin and red blood count are exceptionally low.

Boycott found that in ankylostomatous anæmia the total volume of the blood was increased by 100 per cent., although there was no diminution in the total amount of hæmoglobin. He suggested that the decreased quantity of hæmoglobin per corpuscle may compensate for the increased volume of the blood, so as to enable the necessary oxygenation. During recovery the number of red cells is increased beyond the normal. The pathology of the condition is not distinctly related either to hæmolysis or to hemorrhage; it must be attributed to some alteration of the balance between the in- and outside of the vascular paths.

Ferguson drew attention to the extensive loss of blood from the large number of bites upon the intestinal mucosa. He considered that a large proportion of the bitten areas became septic, and that the products of the sepsis were responsible for some of the symptoms.

Gulland related two cases suggestive of alimentary tract infection which were decidedly improved by the continued administration of fresh uncooked bone marrow. The marrow was obtained from the shin bone of the ox, and one ounce, or more, spread upon thin slices of bread was given three times a day. At first the raw marrow may occasion retching, but this sensation disappears after a few doses. Gulland suggests that the action of the bone marrow is to neutralise the active toxic agents, and that in pernicious anæmia the bone marrow is deficient in this regard, and so succumbs to the presence of the toxin.

The sum total of the discussion does not indicate any new forms of treatment, although it points to a number of problems for investigation, and emphasises the great loss to the community when a case of pernicious anæmia is allowed to pass through our hands without sufficient record of the observations demanded.

To listen to a discussion of this kind is a depressing experience. The needs for investigation are fully evident; the objects to be aimed at are outlined clearly; the possible results are fraught with enormous benefits to the public; yet the slow progress towards fuller knowledge is appalling. The work needs special training in methods of technique, unlimited time for observation and reflection, and financial assistance to adequately maintain those men—and their number should be a large one—who elect to devote their lives to this end. The blanks in our English system of education are perhaps most fully illustrated when the scholar becomes a wage-earner, an elector, or an elected representative; for it is our savage consideration for the care of swine and other animals, rather than that of children, or human adults, which permits the scorn of neighbouring nations and allies; they concern themselves with the care of the pig only so far as its presence or consumption affects the health of the people, but consider it a national duty to promote and finance every possible effort towards the prevention of human disease. For tangible hospital buildings, preferably new ones, for elaborate municipal decorations, for additional libraries and displays of arts, the present generation expends its energies. Would that some men followed, or even surpassed, the example of American and German citizens in their provision for the improved health of the nation! The man of foresight must surely recognise that the national physique is the first line of defence of our country, and that if we are to retain our monumental buildings and our prestige, the present racial degeneration and ill-health must be stopped. In plain words, this means financial support for investigation, and the profession must speak with definite and emphatic voice on this point. The public will respond when we honestly state our case, and logically set forth its requirements. Then when workers are numerous, and Institutes for the investigation of disease are provided, the nation as a whole will become keen upon scientific medicine, and it will not be necessary to deplore the slow progress towards the solution of the problem of this grave anæmia, which at present resists the applications of our limited knowledge.

I. Walker Hall.

